# Electrically controlled variation of receptor affinity

**DOI:** 10.1007/s00216-016-9751-1

**Published:** 2016-07-20

**Authors:** Yulia Efremenko, Vladimir M. Mirsky

**Affiliations:** Department of Nanobiotechnology, Institute of Biotechnology, Brandenburgische Technische Universität Cottbus - Senftenberg, 01968 Senftenberg, Germany

**Keywords:** Chemical sensor, Affinity, Chemotransistor, Conducting polymer, Polyaniline

## Abstract

A concept of virtual sensor array based on electrically controlled variation of affinity properties of the receptor layer is described. It was realized on the base of integrated electrochemical chemotransistor containing polyaniline as the receptor layer. Electrical control of the redox state of polyaniline was performed in five-electrode configuration containing four electrodes for conductivity measurements and one Ag/AgCl reference electrode. All the electrodes were integrated on the same glass chip. A room-temperature ionic liquid was used for the electrical connection between the reference electrode and chemosensitive material. Conductivity measurements demonstrated effective potential-controlled electrochemical conversions of the receptor material between different redox states. Binding of trimethylamine at three different potentials, corresponding to the different states of the receptor material, was studied. Concentration dependencies and binding kinetics were analyzed. The results demonstrated that the kinetic as well as the equilibrium binding properties of the receptor layer can be controlled by electrical potential, thus providing a possibility to form a virtual sensor array using only a single sensing element.

**Graphical abstract:**
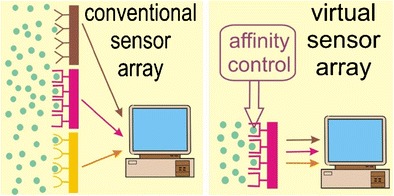
Single sensing element with electrical control of its affinity can operate as a virtual sensor array

Single sensing element with electrical control of its affinity can operate as a virtual sensor array

## Introduction

Chemical compounds applied as receptors in chemical sensors should fulfill a number of requirements. An application for detection of low concentrations of analyte requires a high value of affinity constant. A fast sensor recovery is possible if the kinetic desorption constant of the bond analyte is high enough. Therefore, taking into account that the binding constant is the ratio of kinetic constants of adsorption and desorption, it can be concluded that the binding energy cannot be increased by selection of materials with a higher kinetic desorption constant.

An additional prerequisite is the high selectivity of the analyte binding. In general, it is also defined by the affinity, and receptors with a higher affinity provide a higher selectivity. Despite intensive work in this field [1, 2] that is essentially supported by a wide application of combinatorial and high-throughput techniques [3, 4] and a large number of different compounds which have been synthesized for this purpose (some of affinity properties are reviewed in [5]), it is still difficult to find a selective receptor for a particular application. Therefore, a number of low selective receptors are combined into arrays to improve the selectivity of the whole system. The affinity properties of single sensing elements in the array should be very different (ideally—orthogonal), but a high binding selectivity is not required. Such approach can be relatively easily implemented into the most optical sensors, which enable very simple parallelization of single sensing elements. But in the case of conductometric or electrochemical sensors, an increase of the number of these elements leads to a strong increase in the complexity of the whole sensor system. This is not only a large hindrance in the application of this perspective approach but also a motivation to find a more technological solution.

Earlier, we have reported that the problem of poor recovery of highly sensitive chemical sensors can be solved by application of electrochemical transistors [6, 7]. In this work, we describe an extension of this approach to the formation of virtual arrays of chemical sensors. This novel type of chemical sensors was realized on the basis of electrochemical chemotransistor containing polyaniline (PANI) as the receptor layer. It is a well-characterized chemosensitive material [8]. An interaction of polyaniline with different analytes leads to essential changes in its physical properties which can be detected using different methods. The highest signal-to-noise ratio for transducing the binding of analytes to polyaniline is observed for conductivity measurements [6, 9, 10]. Different measurement configurations were suggested [6]. Simultaneous application of two- and four-point measurements (S_24_ technology) allows one to make independent measurements of bulk and contact resistance, thus providing an internal sensor integrity control. A performance of the conductometric transduction was extended using additional electrodes controlling the redox state of the conducting polymer [6, 7, 11–13]. This configuration, designed as an electrochemical transistor, was further upgraded by the implementation of a S_24_ technique [10], allowing us to combine its advantages with internal integrity control [6].

The suggested approach can be applied for different receptors and for different analytes. In the current work, we use PANI as the receptor and trimethylamine (TMA) as the analyte. TMA is practically important in chemical analytics as one of the main indicators of the spoilage of fish products. Applications of PANI as a receptor for this analyte were earlier applied in chemical sensors based on quartz microbalance [14]. An implementation of the technology of integrated electrochemical transistors allowed us to reach a very high sensitivity and to demonstrate electrically controlled variations of sensor affinity.

We describe here a novel concept for chemical sensors based on conducting polymers: a virtual sensor array consisting of a single chemosensitive element which can be electrochemically converted into materials with different affinity properties. This approach demonstrates that an application of electrochemical chemotransistors can be considered as a possible solution not only for acceleration of sensor recovery [7] but also for a decrease of complexity of sensor arrays.

## Materials and methods

Aniline, trimethylamine, trihexyltetradecylphosphonium chloride (THTDPhCl), sulfuric acid, and AgNO_3_ were purchased from Sigma-Aldrich, and polyacrylic acid was from Fluka. All solutions were prepared with deionized water additionally purified by an EGLA classic system. The sensor chip was fabricated by sputtering of 150-nm-thick gold structures on a Ti/W adhesion layer on a glass wafer. The measurement electrodes used for analysis of bulk and contact resistances have a shape of four parallel strips of 35 μm width separated by 8-μm gaps. The electrode was formed as 300-μm strips around the working electrodes and separated by a 150-μm gap. Before modification, the electrodes were cleaned by acetone, ethanol, water, and piranha solution (mixture of 30 % H_2_O_2_/concentrated H_2_SO_4_, 1:3 (*v*/*v*). *Caution: this solution reacts violently with most organic materials and must be handled with extreme care*), rinsed with water, and dried. The reference Ag/AgCl electrode was fabricated by galvanostatic deposition of silver from aqueous solution of 10 mM AgNO_3_, 20 mM EDTA, 120 mM NH_4_OH, and 80 mM NaOH on the corresponding gold strip at −5 μA for 1800 s followed by an electrochemical formation of AgCl layer in aqueous solution of 0.1 M HCl at 5 μA for 900 s. The inner gold electrodes were coated by PANI, and it was performed by electropolymerization from 0.5 M solution of aniline in 0.5 M aqueous solution of sulfuric acid at +0.9 V vs. SCE. The amounts of the polymer deposited on the measurement electrodes correspond to the polymerization charge of 2.5 mC. Then, the chip surface was covered by a thin layer of trihexyltetradecylphosphonium chloride mixed in 1:1 (*v*/*v*) ratio with 1 % aqueous solution of polyacrylic acid (∼450 kD); an excess of the liquid was removed. The prepared sensors were stable for at least 2 weeks at room temperature. The measurements were performed in the chamber with the volume of 15 mL. To study chemosensitive properties, 0.1 mL of 4.2 M TMA solution in ethanol was injected into a 100-mL narrow-neck flask at room temperature. In ∼30 min when TMA was evaporated, a defined volume of TMA vapor was sucked by a syringe and injected into the sensor chamber.

The measurements were performed at fixed potentials; therefore, it was possible to simplify the wiring of the electrochemical transistor described in [7] and to exclude the auxiliary electrode. Therefore, we used only five electrodes: four (inner) measurement electrodes surrounded by a larger reference electrode. The layer of silver chloride formed on the Ag/AgCl reference electrode operated as a charge buffer for redox conversions of the PANI layer. The technology of simultaneous two- and four-point resistance measurements (S_24_) was described in [10]. Data analysis and extraction of binding constant and kinetic constants of adsorption and desorption was performed according to usual approaches reviewed in [15].

## Results and discussion

In the original design of integrated electrochemical chemotransistor [7], we used a water-based gel electrolytes or water-impregnated alternative layers of cationic and anionic polyelectrolytes. However, the sensor stability was limited by water evaporation. Here, we connected the layer of silver chloride of Ag/AgCl reference electrode with a PANI layer using ionic liquid. After comparison of different ionic liquids, THTDPhCl was selected. It possesses a constant activity of chloride ions, thus providing a constant potential of Ag/AgCl electrode and low viscosity, and is stable in the presence of water. The fabricated chemotransistors coated by THTDPhCl displayed a very low electrical conductivity: depending on the applied potential, the value of the electrical resistance was 3–10 MΩ. A treatment with HCl led to the decrease of the PANI resistance for about 3 orders of magnitude. However, stable chemical sensors can be obtained only by using non-volatile doping compounds. Therefore, instead of HCl, polyacrylic acid was introduced into the electrolyte connecting polyaniline layer with the reference electrode. Because of low solubility in THTDPhCl, it was introduced as an aqueous solution. Further evaporation of water and subsequent increase of chloride concentration (maximum twice) can lead to the shift of the electrode potential for not more than 18 mV; this is an ignorable effect in comparison with the voltage scale required to switch between different redox states of PANI.

**Fig. 1:**
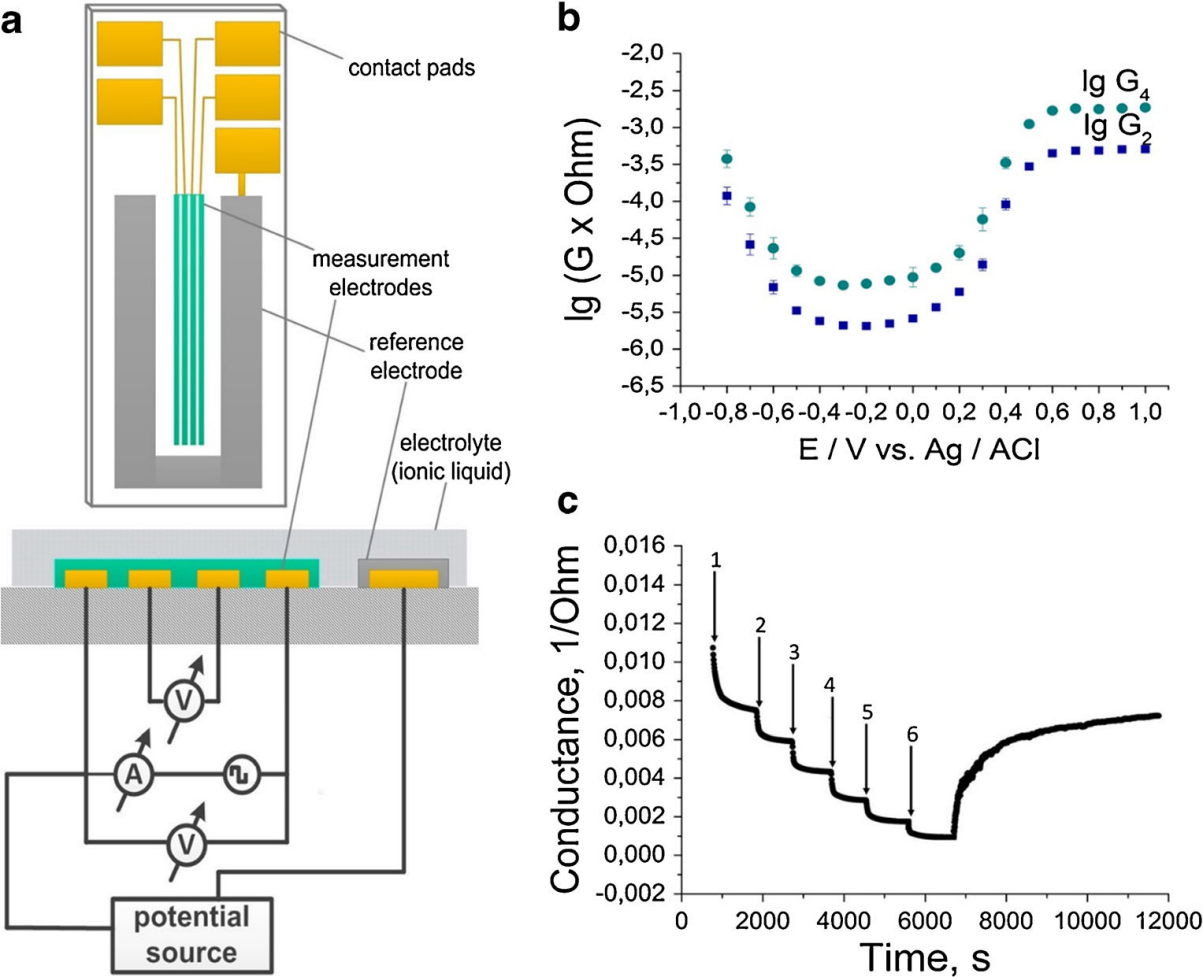
Design and operation of the electrochemical chemotransistor. (**a**) The transistor is based on five-electrode configuration: four inner electrodes serve as working electrodes and are coated by polyaniline, and the outer strip forming a Ag/AgCl reference electrode is connected to the polyaniline coating by a thin layer of chloride-containing ionic liquid. (**b**) Electrical control of the conductance of polyaniline layer measured by four-point (*circles*) and two-point (*squares*) techniques. (**c**) Changes of the sensor signal (conductance measured by a four-point technique) due to subsequent injections of trimethylamine in the concentration of 1 – 0.81 ppm, 2 – 1.62 ppm, 3 – 3.26 ppm, 4 – 6.5 ppm, 5 – 13.0 ppm, and 6 – 26.0 ppm followed by an incubation in pure air

A functionality of the device as a transistor was studied by investigation of the influence of the potential difference between the reference electrode and low-potential outer measurement electrode (further referred as the gate potential) on the resistance of PANI layer. The results are presented in Fig. 1B. The obtained dependence corresponds to that reported in [6, 7] for electrochemical chemotransistors with aqueous gel electrolyte. The data demonstrate electrically controlled conversion of the polymer between three redox states. The first (reduced) state corresponds to the gate potential below −0.8 V. At the gate potential of −0.2 V, PANI is converted to another state. Further oxidation of PANI leads to its third oxidation state observed at the value of gate potential over +0.6 V. These states correspond probably to leucoemeraldine, emeraldine, and pernigraniline [8]. One can imagine that the three states of PANI have different affinity properties; therefore, these three potential values were used for electrochemical modification of sensor properties.

**Fig. 2:**
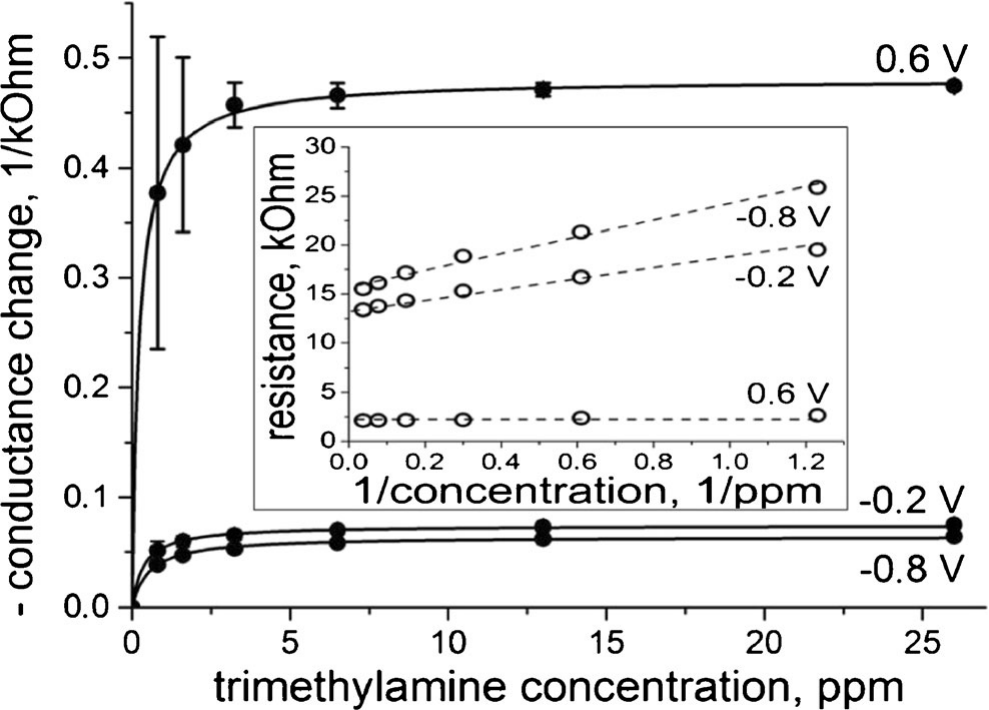
Concentration dependence of the sensor signal for different gate potentials. At high analyte concentrations, the statistical error is smaller than the size of the *symbols. Inset* the same dependences in double reciprocal coordinates

Introduction of TMA into the measurement cell leads to the decrease of the sensor conductance (Fig. 1C). Depending on the concentration of TMA, the effect develops within seconds or minutes and leads to the constant conductance value. Sensor incubation in pure air results in the recovery of the initial sensor conductance within few minutes. Concentration dependence of the sensor signal is shown in Fig. 2. The concentration increase results in monotonous changes of the sensor signal. At high concentrations, this dependence reaches a saturation value which depends on the gate potential. This indicates that the properties of the sensor material at different potentials are different. Oxidation of PANI leads to a higher value of the sensor signal. The obtained concentration dependencies were fitted by Langmuir adsorption isotherms (continues curves in Fig. 2). The good fitting was confirmed by linearization in double reciprocal coordinates (Fig. 2, inset).

The fact that the signal vs. concentration dependence obeys Langmuir adsorption isotherms allows us to suggest that the sensor signal is proportional to the amount of the bond analyte. Using linear extrapolation to a zero value of reciprocal concentration and to a zero value of reciprocal signal [15], the signal values at sensor saturation and the values of the binding constants at different gate potentials were calculated (Table 1). The magnitude of the sensor response increases strongly after the increase of the gate potential from −0.2 V to 0.6 V. This corresponds to the electrochemical conversion of the sensor material into a highly conductive state. This state can provide a higher effect due to analyte-driven conversion into a low-conducting stage. This conducting form of PANI has also a higher affinity to the analyte. The value of the binding energy calculated from the affinity constants increases for ∼2.1 kJ mol^−1^ for the potential change from −0.8 V to +0.6 V. Kinetic desorption constants, extracted from the dependence of signal kinetics on analyte concentrations, demonstrate that the oxidation of the sensor material leads to an increase of the desorption rate for about two times. An extraction of the kinetic adsorption constants from signal kinetics was complicated by too high data scattering; therefore, these values were calculated from the binding constants and kinetic desorption constants. The conversion of the reduced PANI into each next redox state leads to about two times increase of the kinetic adsorption constant. Analytical sensitivity of the sensor response was calculated for small analyte concentrations from the binding constant and maximal sensor signal. Modification of the gate potential leads to over 15 times change of this value.

**Table 1:** Influence of the gate potential on the kinetic adsorption and desorption constants, binding constant, maximal sensor signal, and analytical sensitivity

Gate potential (V)	Adsorption kinetic constant, ppm^−1^ s^−1^	Desorption kinetic constant, s^−1^	Binding constant, ppm^−1^	Maximal sensor signal, Ω^−1^	Analytical sensitivity, Ω^−1^ × ppm^−1^
−0.8	6.8 × 10^−4^	4.5 × 10^−3^	1.52	6.4 × 10^−2^	9.7 × 10^−2^
	±3.8 × 10^−4^	±3 × 10^−5^	±4 × 10^−2^	±2 × 10^−3^	±4 × 10^−3^
−0.2	1.8 × 10^−2^	6.5 × 10^−3^	2.73	7.6 × 10^−2^	0.21
	±5 × 10^−4^	±5 × 10^−5^	±5 × 10^−2^	±4 × 10^−3^	±1 × 10^−2^
+0.6	3.0 × 10^−2^	8.0 × 10^−3^	3.76	4.5 × 10^−1^	1.6
	±2 × 10^−3^	±2 × 10^−4^	±2 × 10^−2^	±5 × 10^−2^	±7 × 10^−2^

## Conclusion

We have developed a new concept for electrical control chemosensitive properties. The concept allows one to modify the affinity properties of chemical receptors enabling a reduction of a number of sensing elements in sensor arrays. One can imagine even a virtual *array* consisting of a single sensing element whose affinity properties are switched by electrically driven oxidation or reduction: instead of using of few sensors containing different chemosensitive materials, the signals of the same chemosensitive material at different potentials are measured.

In this work, an electrical control of the sensor affinity was implemented and studied using integrated electrochemical chemotransistors with PANI as a chemosensitive element. Multifold variations of the binding constant and of the kinetic adsorption and desorption constants were observed. But the suggested approach can be considered more generally; it can be probably realized by using not only conducting polymers but also other types of redox-active chemosensitive materials.
